# Genomic profiling of sporadic multiple meningiomas

**DOI:** 10.1186/s12920-022-01258-0

**Published:** 2022-05-14

**Authors:** E. Zeynep Erson-Omay, Shaurey Vetsa, Sagar Vasandani, Tanyeri Barak, Arushii Nadar, Neelan Marianayanam, Kanat Yalcin, Danielle Miyagishima, Stephanie Marie Aguilera, Stephanie Robert, Ketu Mishra-Gorur, Robert K. Fulbright, Declan McGuone, Murat Günel, Jennifer Moliterno

**Affiliations:** 1 Department of Neurosurgery, Yale School of Medicine, 15 York St, LLCI 810, New Haven, CT 06520-8082 USA; 2 The Chênevert Family Brain Tumor Center, Smilow Cancer Hospital, New Haven, CT USA; 3 The Susan Beris, MD Neurosurgical Oncology Program at Yale New Haven Hospital, New Haven, CT USA; 4 Department of Radiology and Biomedical Imaging, Neuroradiology Section, Yale School of Medicine, New Haven, CT USA; 5 Department of Pathology, Yale School of Medicine, New Haven, CT USA; 6 Department of Genetics, Yale School of Medicine, New Haven, CT USA

**Keywords:** Genomics, Sporadic multiple meningiomas, Clonal formation

## Abstract

**Background:**

Multiple meningiomas (MMs) rarely occur sporadically. It is unclear whether each individual tumor in a single patient behaves similarly. Moreover, the molecular mechanisms underlying the formation of sporadic MMs and clonal formation etiology of these tumors are poorly understood.

**Methods:**

Patients with spatially separated MMs without prior radiation exposure or a family history who underwent surgical resection of at least two meningiomas were included. Unbiased, comprehensive next generation sequencing was performed, and relevant clinical data was analyzed.

**Results:**

Fifteen meningiomas and one dural specimen from six patients were included. The majority of tumors (12/15) were WHO Grade I; one patient had bilateral MMs, one of which was Grade II, while the other was Grade I. We found 11/15 of our cohort specimens were of *NF2*-loss subtype. Meningiomas from 5/6 patients had a monoclonal origin, with the tumor from the remaining patient showing evidence for independent clonal formation. We identified a novel case of non-*NF2* mutant MM with monoclonal etiology. MMs due to a monoclonal origin did not always display a homogenous genomic profile, but rather exhibited heterogeneity due to branching evolution.

**Conclusions:**

Both *NF2-*loss and non-*NF2* driven MMs can form due to monoclonal expansion and those tumors can acquire inter-tumoral heterogeneity through branched evolution. Grade I and II meningiomas can occur in the same patient. Thus, the molecular make-up and clinical behavior of one tumor in MMs, cannot reliably lend insight into that of the others and suggests the clinical management strategy for MMs should be tailored individually.

**Supplementary Information:**

The online version contains supplementary material available at 10.1186/s12920-022-01258-0.

## Background

Meningioma is the most common primary intracranial tumor and arises from the meninges covering the brain and spinal cord. Approximately 10% of all meningiomas occur as multiple tumors, either as at least two lesions clustering near one another, or “synchronously,” or spatially separated without an anatomical bridge, or “metachronously” [1]. Multiple meningiomas (MMs) can be seen in both familial and sporadic forms, as well as in radiation induced cases that are associated with a more aggressive clinical course and higher risk of recurrence [1, 2]. Current standard of care for meningiomas includes maximal safe surgical resection for tumors that are growing or causing symptomology. However, the multiplicity of meningiomas might present challenges regarding the prioritization and the need for surgical resection. While it has been reported that approximately one-third of all meningiomas receive treatment, almost two-thirds of MMs require intervention, suggesting more aggressive clinical behavior associated with multiple tumors irrespective of radiation exposure [1]. Therefore, an understanding of the genomic characterization of sporadic MMs could be useful to guide treatment decisions aimed at improving patient outcomes.

The genomic basis of ~ 80% of WHO Grade I meningiomas has been well described with somatic mutations affecting *NF2, TRAF7, AKT1, KLF4, SMO, SUFU, POLR2A and PI3K* pathway genes, along with copy number alterations, the latter of which are especially prevalent in higher grade tumors [3–7]. The genomics of familial MMs is also well understood with known inherited driver germline mutations in *NF2, SUFU*, or SWI/SNF complex (*SMARCE1, SMARCB1*) genes [8–10]. While the molecular basis for the formation and development of sporadic MMs has been suggested to be through either mono- or multi-clonal (i.e., independent from one another) formation [11, 12], the genomic studies to test these hypotheses have been limited [13–17].

Herein we present the genomic analysis of a cohort of sporadic MMs and to our knowledge, as the first study in which whole exome sequencing (WES) is performed and somatic single nucleotide variation (SNV), small insertion/deletion (INDEL) events and copy number variations (CNV) are analyzed along with robust clinical data.

## Methods

The study protocol was approved by the Yale Human Investigation Committee (HIC). All patients who underwent surgical resection by a single neurosurgeon (JM) of histologically and radiologically confirmed MMs at Yale New Haven Hospital with available somatic genomic data were considered for this study. Patients with radiation-induced MMs or those with a germline mutation potentially related to meningioma formation were excluded. The details for study exclusion criteria can be found in Additional file 1: Fig. S1.

### Radiographic classification of multiplicity

Pre- and postoperative magnetic resonance imaging (MRI) and computed tomography (CT) imaging were evaluated for each patient by a board certified neuroradiologist (RKF). Classification of location was determined as we have previously described before[18, 19]. Extent of resection (EOR) was defined using Simpson Grading [20], with Simpson grades 1–3 being considered gross total resection (GTR). Proximity of one meningioma to another in an individual patient with MMs was determined by measuring the distance (in millimeters) between the centroids of two meningiomas, i.e., center-to-center. An edge-to-edge distance was also measured, which was defined as the minimum distance between the edges of two meningiomas. While the edge-to-edge metric indicates maximal closeness, the center-to-center distance represents an average distance. These distance calculations were made between all MMs in each patient.

### Tumor pathology

Histological subtypes and the World Health Organization (WHO 2016) Grade of all tumors was determined by board-certified neuropathologists. Images were captured on an Olympus BX40 camera using spot idea CMOS camera and software. The final image resolution of 200 × is obtained for all histological images, by 10 × optical zoom together with 20 ×  of lens magnification. No enhancements were used.

### Genomic analysis and subgroup classification of multiple meningiomas

Genomic DNA from the tumor and dura samples, along with the matching blood from all cases, was extracted and coding regions were captured using xGEN Exome Research Panel v1.0 kit (Integrated DNA Technologies, Coralville IA) with the additional spike-in regions. Sequencing was performed at the Yale Center for Genome Analysis (YCGA) on Illumina NovaSeq6000 platform with 2 × 100 bp reads yielding high coverage (Additional file 2: Table S1). Downstream analysis was performed using GATK (v4.1.9, Grch37) as described before [21].

GATK Mutect2 (v4.1.9) was used to call somatic SNV/INDELs with the default parameters in tumor-matched normal mode, and with gnomAD as the germline resource (Mutect2 resource bundle). FilterMutectCalls was used to exclude the variants with variant allele frequency (VAF) less than the calculated contamination rate. Annotation was done using Funcotator (dataSources.v1.7.20200521s) with the “BEST_EFFECT” transcript-selection-mode. Downstream filtering was performed by excluding variants with allele frequency greater than 0.01 in gnomAD-genome data (v2.1) and by keeping variants with functional coding impacts. Somatic CNVs were called using GATK-Somatic CNV calling pipeline with matching normal data using the “minimum interval median percentile” parameter set to 5.0 and other parameters set to default values.

Classification of meningiomas based on genomic profile was carried out as previously described [18, 19]. Tumors with somatic *NF2* mutation and/or a chromosome 22 deletion, were classified as “*NF2*-loss” molecular subtype, whereas tumors with other drivers were grouped as “non-NF2” subtype.

### Phylogeny analysis

A binary matrix was formed for somatic SNV/INDEL and CNV calls including all lesions for every sample. Somatic SNV/INDELs involving the meningioma related genes were used (*NF2, TRAF7, AKT1, KLF4, PIK3CA, PIK3R1, SMO, SUFU, POLR2A, SMARCB1,* and *PRKAR1A*) [19].

We have used the allele-fraction model-based segmentation generated by the GATK-Somatic-CNV pipeline. Shared segments among lesions of the same sample were identified using “intersect” method in bedtools (v2.29.2). The distance matrix was created using the manhattan distance. Neighbor-joining tree estimation from APE R package (5.4.1) was used to create and plot the phylogeny model of the lesions from the calculated distance matrix [22].

### List of reagents/kits

Ki-67 staining is performed on the Leica Bond-III platform using Bond Refine Detection (catalog number = DS9800) with Epitope Retrieval Solution 2 (catalog number: AR9640). Antibody is purchased from DAKO (clone: MIB-1, catalog number: M7240) and dilution factor of 1:300 is used.

For H&E and PAS staining: All materials are from Polyscientific R & D. Harris Hematoxylin with Glacial Acetic Acid (catalog number: S212A). Eosin Y Alcoholic (catalog number: S2186); and Schiff Reagent for PAS (catalog number: S27).

## Results

### Cohort characteristics

**Fig. 1:**
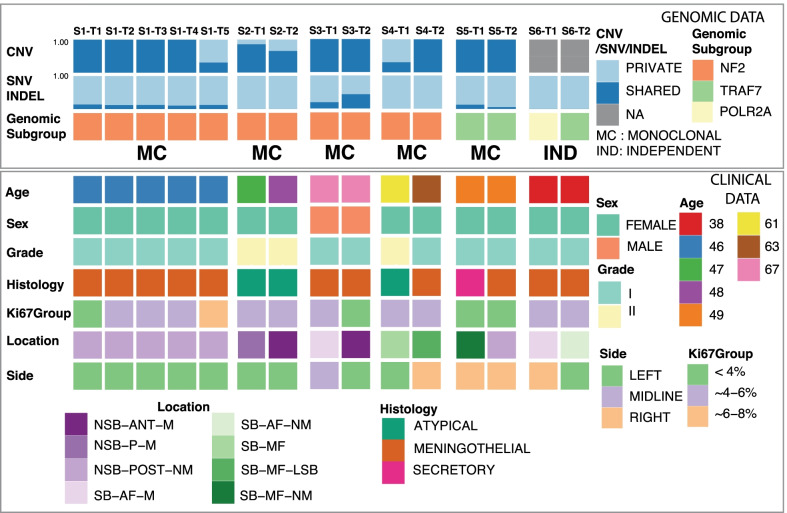
Schematic summary of the study cohort. First top two panels represent the fraction of private and shared somatic alterations among the lesions of the same patient, CNVs and SNV/INDELs, respectively. Third panel annotation belongs to genomic subgroup with the clonal formation pattern concluded with the study, i.e. MC: Monoclonal formation, IND: Independent formation. Lower group panels summarize the clinical, histological and genomic attributes, such as age, sex, grade, histology, Ki-67 classification and location. SB: Skull Base, NSB: Non-Skull Base, AF: Anterior Fossa, MF: Middle Fossa, M: Midline, NM: Non-Midline, ANT: Anterior, POST: Posterior

Our cohort consisted of one “uninvolved” dural and 15 tumor specimens from six individuals; five of these patients each underwent resection of two meningiomas, three of them still having at least 2 unresected tumor. One patient had five lesions, all of which were removed. We observed a female preponderance (5/6) and median age of 48 years (Table 1, Fig. 1). All tumors were metachronous with no anatomical bridging in between tumors. The average center-to-center and edge-to-edge distances between MMs in patients were 53.1 mm and 33.7 mm, respectively (Table 1). Whereas MMs in four of the patients were resected during the same surgery, two patients each underwent two separate surgeries for removal of their tumors given the increased distance. All patients underwent GTR of their MMs (Additional file 2: Table S1) There were 12/15 cases with complete resection and removal of associated bone and dura (Grade I) and 3/15 cases with complete resection of the tumor and coagulation of underlying dura (Grade II) on the Simpson Grading scale. The majority of tumors (12/15) were WHO Grade I, with the remaining being Grade II. One patient had two meningiomas, one WHO Grade I and the other Grade II. No patients experienced recurrence during this study (median follow up = 7.7 months).

**Table 1:** Clinical characteristics of the cohort. Ki-67 ≥  5% was defined as high proliferative index while < 5% was defined as low proliferative index

Characteristics	Count/median (range)	Percentage
Total number of patients	6	100%
Age at first surgery (years)	48	
Sex		
Female	5	83.3%
Male	1	16.7%
Race		
Caucasian	5	83.3%
Black	1	16.7%
Volume (cm^3^)	2.63	
Ki-67 index		
Low	9	60%
High	6	40%
Intracranial laterality		
Unilateral	1	16.7%
Bilateral	5	83.3%
WHO grade		
I	12	80%
II	3	20%
Extent of resection (Simpson grade)		
I	12	80%
II	3	20%
Recurrence		
Yes	0	0%
No	15	100%
Intertumoral distance (mm)		
Edge-to-edge	25.1 (3.1–90.0)	
Center-to-center	48.9 (20.1–122.3)

### Clonality insight and subgrouping

The analysis of the somatic SNV/INDEL and CNV data revealed that MMs in five of the six patients had a monoclonal origin, while the remaining one displayed a multi-clonal, independent pattern. Eleven tumors from four individuals (S1-T1-T5, S2-T1-T2, S3-T1-T2, S4-T1-T2) were of “*NF2*-loss” molecular subtype. Three tumors from two individuals (S5-T1-T2, S6-T2) had somatic *TRAF7* mutations and one tumor (S6-T1) harbored a somatic *POLR2A* mutation (Fig. 1).

Consistent with the previous literature [11, 12, 17], all *NF2*-loss MMs displayed a monoclonal origin (S1, S2, S3, S4). We also identified one patient (S5) with two non-*NF2* mutant tumors displaying a monoclonal origin. One patient, S6, with two non-*NF2* tumors was classified as multi-clonal.

### NF2-loss MMs of monoclonal origin

**Fig. 2:**
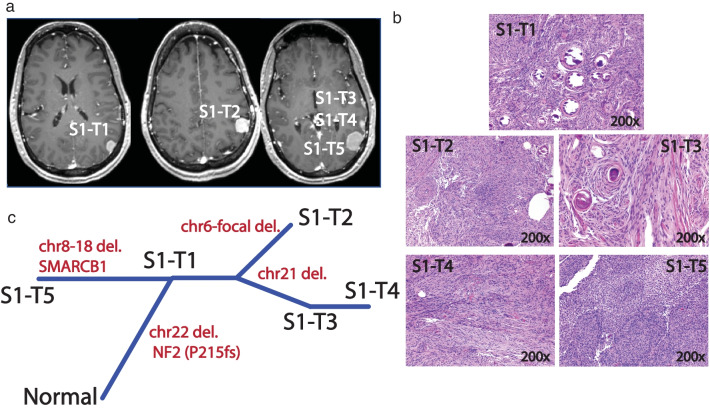
Representation of case S1. **a** MRI of 5 lesions, **b** hematoxylin and eosin (H&E) stain (magnification: 200×) for all 5 lesions, S1-T3: meningioma with psammomatous calcifications. **c** Phylogeny inferred from the somatic CNV, SNV/INDEL data of the lesions displaying monoclonal formation and branched evolution

In one of the monoclonal *NF2*-loss MMs cases (S1), all five tumors were of the meningothelial histological subtype and all shared chromosome 22 deletion and the same *NF2* mutation. Somatic analysis of a corresponding uninvolved dura sample did not reveal any somatic alterations previously reported in meningiomas. One tumor in this sample, S1-T5, harbored a *SMARCB1*: p.R377H mutation and additional CNV events on chromosomes 8 and 18 (Fig. 2). Overall, these analyses revealed a branched evolution pattern (phylogeny shown in Fig. 2c).

**Fig. 3:**
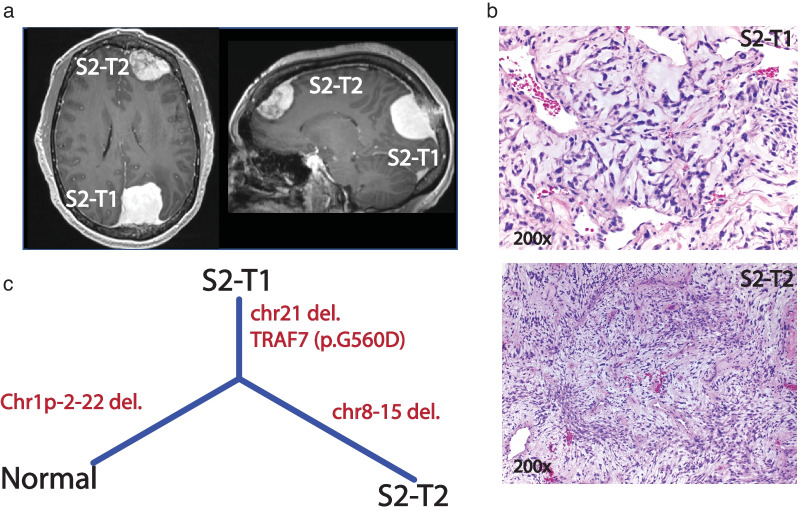
Representation of case S2. **a** MRI of the frontal (S2-T2) and the occipital lesion(S2-T1), **b** hematoxylin and eosin (H&E) stain (magnification: 200×) for both Grade II lesions, S2-T1: with chordoid features marked with black arrows. **c** Phylogeny inferred from the somatic CNV, SNV/INDEL of the 2 lesions

We observed a similar branching evolution pattern in another patient (S2). The two meningiomas, one frontal and one occipital, harbored a somatic *NF2* mutation along with deletions affecting chromosomes 1p, 2 and 22, suggesting a monoclonal origin (Fig. 3). However, while the occipital meningioma (S2-T1) acquired a new somatic mutation in TRAF7: p.G560D, which was previously reported in meningiomas [3], the frontal tumor (S2-T2) acquired additional CNV events affecting chromosomes 8 and 15. Both meningiomas were atypical, WHO Grade II. In another patient (S3) with two meningiomas, the two tumors shared the same CNV events involving multiple chromosomes, including chromosome 22 deletion, suggesting monoclonal origination (Additional file 3: Fig. S2a). While one of the meningiomas was located along the convexity, the other localized to the skull base, a region usually associated with non-*NF2* mutated meningiomas [3, 18]. In another case with a monoclonal formation, a founding clone harboring a driver CNV event (chr22 deletion), later evolved into two tumors by acquiring two distinct driver mutations, *NF2*: p.Q410X and *NF2*: p. F62fs, in S4-T1 and S4-T2, respectively (Additional file 3: Fig. 2b). Our CNV analysis also revealed that S4-T1 acquired additional CNV events including chr1p deletion, which has been previously reported in higher grade meningiomas [6]. Indeed, S4-T1 tumor was diagnosed as WHO Grade II with atypical histology, whereas S4-T2 was Grade I and meningothelial subtype. Interestingly, these two tumors (S4-T1 and S4-T2) were located bilaterally with a distance greater than the average distance observed between multiple tumors in the cohort (76.4 mm vs. 53.12 mm, Additional file 2: Table S1).

### Non-*NF2* MMs of monoclonal origin

**Fig. 4:**
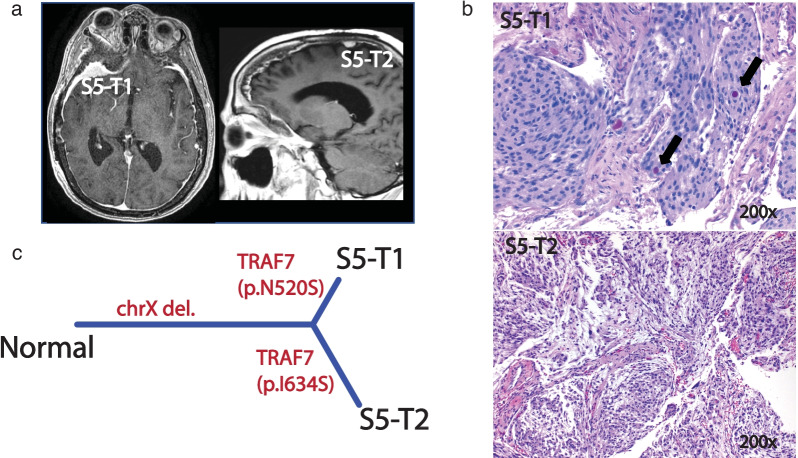
Representation of case S5. **a** MRI of 2 lesions, **b** hematoxylin and eosin (H&E) stain (magnification: 200×) for both lesions, S5-T1: secretory meningioma with periodic acid Schiff positive pseudopsammoma bodies, black arrows. **c** Phylogeny inferred from the somatic CNV, SNV/INDEL of the 2 lesions

We demonstrated the monoclonal origin of two non-*NF2* mutated meningiomas in another patient (S5), with a right sphenoid wing and right convexity tumor. Interestingly, the right sphenoid wing tumor (S5-T1) displayed a secretory subtype which is characterized by foci of gland differentiation with periodic acid Schiff positive eosinophilic globular secretions (Fig. 4b), Both meningiomas shared a large-scale chromosome × deletion but harbored two distinct *TRAF7* mutations (p.N520S and p.I634S) (Fig. 4). In an effort to assess the clonality and order of occurrence of these somatic events, we compared the calculated minor-allele-fraction (MAF) for chromosome X deletion and VAF for the *TRAF7* mutations in both cases. The MAF in the segment with LOH on chromosome X was 0.30 and 0.11, indicating a clonality of 40% and 80% for S5-T1 and S5-T2, respectively. Interestingly VAF for *TRAF7* mutations were 22% and 40%, indicating the same clonality rates of 40% and 80% in S5-T1 and S5-T2.

### Multi-clonal MMs

Finally, in patient (S6), who harbored two tumors, (a right sphenoid wing and a left anterior skull base tumor), we identified completely distinct genomic profiles (Additional file 3: Fig. S2c). Although both tumors exhibited meningothelial histology, they harbored somatic mutations affecting *TRAF7* and *POLR2A,* respectively, revealing distinct molecular mechanisms for formation and a multi-clonal origin despite a seemingly similar histological subtype.

## Discussion

Previous studies investigating the genomics of sporadic MMs have been limited due to single cases [23], or the utilization of limited genomic screening methods, without considering CNV events along with the SNV/INDELs [17]. CNV events, in particular, are known to play a significant role in both the formation and progression of meningiomas and therefore CNVs are essential when assessing the clonal origin of a tumor [3–7].

With the use of comprehensive and unbiased genomic analyses, we demonstrate that sporadic MMs in the same patient can show both genomic and histologic heterogeneity. Though more commonly monoclonal in our cohort, MMs can be of both mono- and multi-clonal origin. Furthermore, we show that monoclonal formation can be observed in both *NF2*-loss and non-*NF2* mutant tumors and those meningiomas can undergo branched evolution to display intertumoral heterogeneity in the same patient (Figs. 2c, 3c, 4c). While it has been previously reported that MMs in the same patient can be of different histological grade [1, 24, 25], we add that this also corresponds to different underlying molecular make-up. Thus, the biology and potential clinical behavior of one tumor, in a patient with MMs, cannot reliably lend insight into that of others.

Interestingly, we found that even when MMs share a monoclonal origin, they can exhibit genomic heterogeneity through branched evolution, with potential clinical implications. For instance, we showed in a patient with five *NF2*-loss meningiomas, one of the tumors acquired the pathogenic *SMARCB1* mutation [3]. *NF2:SMARCB1* co-mutated meningiomas have been shown to have a higher proliferative index [19], are part of the pathway to aggressiveness and higher grade in meningiomas [5] and seem to benefit from greater EOR with regards to prevention of recurrence [19]. Indeed, while this S1-T5 tumor was WHO Grade I, it did have an elevated proliferative index (Ki67 6–8%), while the four other tumors in this individual all displayed low indexes. Resection of one of the other four meningiomas, would not have allowed for this understanding.

Histological and anatomical correlations have been well established for sporadic meningioma and are useful in predicting the underlying genomic driver mutation [18]. However, we have shown that the histological subtype, as well as the intracranial origin of each tumor in a MM patient, can vary and is not always reliable in inferring the genomic landscape of each of the multiple tumors as may be the case in solitary lesions. In another example demonstrating the branched evolution pattern of MMs (S5), we showed that while two tumors shared a chromosome X deletion, they harbored two distinct *TRAF7* mutations along with two different histological subtypes. Interestingly, while the sphenoid wing tumor (S5-T1) was histologically classified as a secretory subtype, the right convexity tumor (S5-T2) was of meningothelial histology, the latter with clinical features being highly unusual for *TRAF7* mutated meningiomas (Fig. 4). A similar finding was observed in patient (S4) with bilateral sphenoid wing meningiomas, in which the tumors were different grades and histologic subtypes, with the higher-grade tumor harboring additionally acquired CNV events. Thus, even in MMs with monoclonal origin, complex evolution patterns can result in genomically distinct tumors with different corresponding clinically relevant characteristics.

Interestingly, the distance between MMs does not seem to confer any useful inferences. In two cases with monoclonal origination and underlying heterogenous genomics, S2 and S4, the MMs were located at two very distant anatomical locations. Indeed, S2 had the largest intertumoral distance between the right occipital tumor and the right frontal tumor compared to the others (Fig. 3). Therefore, even in cases in which the MMs are quite distant and seemingly distinct and potentially “separate” lesions, they can still arise from the same clone. In a similar case with underlying genomic homogeneity, one tumor was located along the medial anterior skull base with the other nearby and abutting the anterior-most aspect of the left superior frontal gyrus (S3), without any obvious diseased dura in between (Additional file 3: Fig. S2). Finally, in the one case of multiclonal origin in a patient (S6), who harbored two tumors, one with a somatic *TRAF7* and another with a *POLR2A* somatic mutation (Additional file 3: Fig. S2) we found the anatomical distance between these two genomically distinct tumors (center-distance = 48.1 mm) to be comparable to the monoclonally originating tumors (mean center-distance = 53.48 mm). These illustrations further emphasize that neither the anatomical proximity of the MMs, nor the molecular make-up and histological evaluation of a single tumor from a patient with MMs, may fully represent the biology of each tumor and may fail to reflect the scope of the overall disease and projection of its evolution**.** Thus, management of MMs should be based on each individual tumor’s clinical behavior (i.e. growth, symptomology).

These observations highlight the complexity of MMs and our lack of understanding of how they form, especially from a monoclonal origin. Different theories have been proposed, including potential dissemination of tumor cells along the cerebrospinal fluid or subarachnoid space [12, 14]. Additionally, it has been postulated that MMs form through dural spread, supported by the radiographic observation of “dural tails” frequently seen with these tumors. However, in all of the monoclonal MMs in our cohort, there was no concern radiographically or intraoperatively for involvement of the bridging dura, and certainly no connection between the tumors in the more distance cases. Moreover, when we analyzed a dural sample, taken from an uninvolved area centrally located to five MMs in one patient, we did not detect any genomic abnormality known to be associated with meningioma formation. Therefore, our findings suggest against dural spread being the explanation for MM formation. In the absence of a germline mutation, however, further investigation is needed to understand the pathogenesis.

### Study limitations

MMs are relatively rare, and this is reflective in the small sample size included in this cohort. However, the use of comprehensive genomic characterization allowed for a more in-depth evaluation of the genomics of these tumors and provides novel insight into clonality. Also, we recognize the median follow-up time of the patients included was relatively short. However, the focus of this study was to provide an understanding of the inter-tumoral heterogeneity in a MM patient and how one tumor cannot reliably represent that of another highlighting clinical implications. Further studies are needed to better understand this relationship with clinical behavior, and specifically longer-term recurrence.

## Conclusion

Using comprehensive genomic profiling techniques, we revealed the heterogeneity of MMs in any given patient such that sporadic MMs can be of both mono- and multi-clonal origin, with both *NF2* and *non-NF2* driven MMs associated with the former. MMs in the same patient can be of different histologic sub-type, grade and molecular make-up. Monoclonal MMs can acquire inter-tumor heterogeneity due to additional somatic alterations through branched evolution. Our findings underscore the importance of unbiased, comprehensive genomic analyses to determine the complex etiology and variability of sporadic MMs and that the pathology and landscape of one tumor may not be representative of the others. We recommend management of MMs in any given patient on an individual basis, with surgery aimed at removing the most number at one time, when feasible.

## Supplementary Information

**Additional file 1.Supplementary Figure 1:** The flow-chart depicting the algorithm for sample selection.

**Additional file 2.** **Supplementary Table 1:** Cohort summary table with sequencing and clinical details.

**Additional file 3.** **Supplementary Figure 2:** MRI, hematoxylin and eosin (H&E) stain (magnification: 200×) and phylogeny inferred from the somatic CNV, SNV/INDEL of the 2 lesions for a. S3, b.S4, c.S6.

## Data Availability

The sequencing data analyzed as part of this data is submitted to EGA under the accession number EGAS00001005700.

## Abbreviations

MMs: Multiple meningiomas
WES: Whole exome sequencing
SNV: Single nucleotide variation
INDEL: Small insertion/deletion events
CNV: Copy number variations
MRI: Magnetic resonance imaging
EOR: Extent of resection
GTR: Gross total resection
MAF: Minor-allele-fraction
H&E: Hematoxylin and eosin
