# Correction: Genomic profiling of sporadic multiple meningiomas

**DOI:** 10.1186/s12920-022-01273-1

**Published:** 2022-06-13

**Authors:** E. Zeynep Erson-Omay, Shaurey Vetsa, Sagar Vasandani, Tanyeri Barak, Arushii Nadar, Neelan J. Marianayagam, Kanat Yalcin, Danielle Miyagishima, Stephanie Marie Aguilera, Stephanie Robert, Ketu Mishra-Gorur, Robert K. Fulbright, Declan McGuone, Murat Günel, Jennifer Moliterno

**Affiliations:** 1 Department of Neurosurgery, Yale School of Medicine, 15 York St, LLCI 810, New Haven, CT 06520-8082 USA; 2 The Chênevert Family Brain Tumor Center, Smilow Cancer Hospital, New Haven, CT USA; 3 The Susan Beris, MD Neurosurgical Oncology Program at Yale New Haven Hospital, New Haven, CT USA; 4 Department of Radiology and Biomedical Imaging, Neuroradiology Section, Yale School of Medicine, New Haven, CT USA; 5 Department of Pathology, Yale School of Medicine, New Haven, CT USA; 6 Department of Genetics, Yale School of Medicine, New Haven, CT USA

## Correction to: BMC Medical Genomics (2022) 15:112 https://doi.org/10.1186/s12920-022-01258-0

Following publication of the original article [1], the authors identified an error in the author name of Neelan Marianayagam.

The incorrect author name is: Neelan Marianayanam.

The correct author name is: Neelan J. Marianayagam.

The author group has been updated above and the original article [1] has been corrected.
