# Supplementary figures and images for: Genomic profiling of sporadic multiple meningiomas
Source: BMC Med Genomics. 2022 May 14;15:112. doi: 10.1186/s12920-022-01258-0 (PMC9107270; doi:10.1186/s12920-022-01258-0)

Supplementary Figure 1

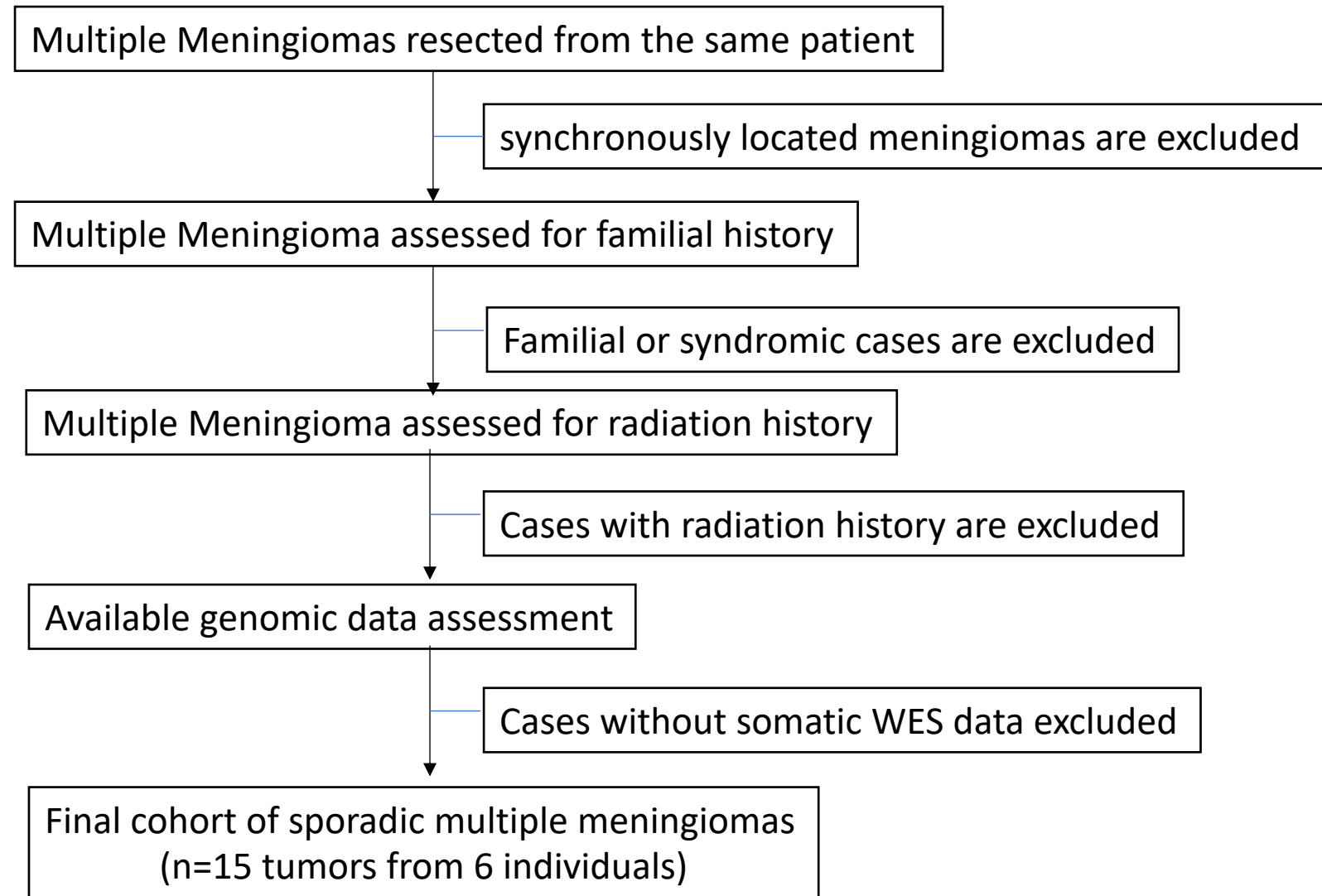

Supplement: Supplementary file 1 — Additional file 1.Supplementary Figure 1: The flow-chart depicting the algorithm for sample selection. [file 12920_2022_1258_MOESM1_ESM.pdf]
